# Solving difficult structures with electron diffraction

**DOI:** 10.1107/S2052252514026797

**Published:** 2015-01-01

**Authors:** J. M. Zuo, J. L. Rouviére

**Affiliations:** a Department of Materials Science and Engineering, University of Illinois, Urbana, IL 61801, USA; b Seitz Materials Research Laboratory, University of Illinois, Urbana, IL 61801, USA; c CEA/INAC/SP2M/LEMMA, 19 rue des Martyrs, Grenoble, 38 054, France

**Keywords:** precession electron diffraction, electron crystallography, electron techniques, electron-based structure analysis

## Abstract

Precession electron diffraction has solved a long-standing challenge in electron diffraction. Further progress promises a general technique for structure determination of difficult crystals.

Electrons diffract in the same way as X-rays and neutrons, except that the electron wavelength is very small (of the order of a few picometers for 80–300 keV electrons), and the electron scattering cross-section is much larger, about a million times that of X-rays. Inside a transmission electron microscope (TEM), the electron beam can be focused down to ~1 Å in diameter with the current reaching hundreds of picoamps (1 pA ≃ 6.3x10^6^ e s^−1^), so the scattering power of an electron beam is larger than that of a synchrotron. Since electron diffraction was discovered by Davisson and Germer, and Thomson and Reid, in 1927, transmission electron diffraction and the related electron imaging have developed into powerful tools for the analysis of defects, microstructure, surfaces and interfaces in a broad range of materials. So why haven’t more unknown crystal structures been solved with high-energy electrons?

The short answer lies in electron dynamic diffraction: the same strong interaction between electrons and matter that gives rise to large electron scattering cross sections also leads to strong multiple scattering. The theory of electron multiple scattering was developed as early as 1928 by Hans Bethe in his remarkable PhD thesis. Electron dynamic diffraction can allow the phase of structure factors to be determined to an accuracy of 0.2° by refining the electron diffraction intensity recorded in a convergent beam electron diffraction (CBED) pattern using the calculated dynamic intensities (Jiang *et al.*, 2010 ▶). However, the refinement method requires a known structure. A general method for solving unknown crystal structures using dynamic diffraction intensities has yet to be developed, despite many outstanding efforts in the past (Spence *et al.*, 1999 ▶; Allen *et al.*, 2000 ▶; Koch, 2005 ▶).

In the topical review by Midgley and Eggeman (Midgley & Eggeman, 2015 ▶), the authors describe the remarkable progress made in an alternative approach to electron structure solution, precession electron diffraction (PED), a technique discovered 20 years ago by Vincent & Midgley (1994 ▶). In PED, the incident electron beam rotates around a crystal direction, keeping a constant angle – the ‘precession angle’ – with this crystal direction. To compensate for the motion of diffracted beams as the incident beam rotates, the outgoing beams are deflected back (Fig. 1 ▶ in Midgley & Eggeman, 2015 ▶), similar to the double rocking technique for the recording of large-angle CBED patterns (Eades, 1980 ▶). By recording electron diffraction patterns with the incident electron beam in precession, PED is able to provide the integrated electron diffraction intensity across the Bragg condition for many reflections. The use of such intensities for structure solution in numerous test structures has shown surprising robustness against crystal thickness variations and small crystal misorientations, which could have a dramatic effect on electron diffraction intensities recorded using conventional techniques (see Fig. 1 ▶). Using PED intensities, crystal structures can be solved by a combination of phasing and structure refinement, where the *R* factor can be reduced to less than 10% by further including dynamic effects (Palatinus *et al.*, 2013 ▶; Jacob *et al.*, 2013 ▶).

Over the past decade, the development of aberration correctors for high-resolution electron microscopes has brought worldwide excitement and tremendous progress in real-space-based structure determinations using atomic resolution imaging and chemical analysis. Applications of these techniques tend to focus on the so-called radiation-hard materials, such as metals and ceramics. Since electron diffraction provides the strongest analytical signal inside a TEM, it can therefore be applied to small and complex (difficult) crystals. With the welcoming developments in PED, and its integration with the data acquisition tools of automated diffraction tomography (ADT, Kolb *et al.*, 2007 ▶), scanning and automated diffraction pattern indexing and analysis (see review in Midgley & Eggeman, 2014 ▶), electron diffraction is rapidly developing into a truly quantitative crystallographic tool for the determination of atomic structure as well as complex microstructures. It is thus heartening to see a broad range of structures, including organic frameworks, complex zeolites, germano–silicate frameworks and organic crystals solved by PED (see Midgley & Eggeman, 2015 ▶).

What is the future for electron diffraction? The quality of electron diffraction data, as well as speed of acquisition, is increasing rapidly with the development of fast cameras, sophisticated beam and sample manipulation methods, and data analysis (Koch, 2011 ▶; Kim & Zuo, 2013 ▶; Kim *et al.*, 2013 ▶). Thus, in a not too distant future, we can expect more identifications of new structures and their solutions, especially in mixed phase materials or at interfaces and grain boundaries. Another intriguing possibility is to combine precession with high-order aberration corrections for precession scanning transmission electron microscopy (PSTEM). By reducing dynamical effects in the electron probe scattering using precession, significant gains can be achieved in quantitative three-dimensional electron imaging as well as chemical analysis.

## Figures and Tables

**Figure 1:**
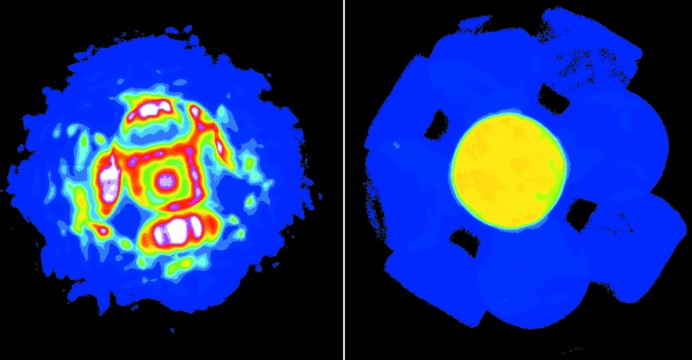
CBED patterns recorded using 200 kV electrons from Si along [001] (left) without and (right) with precession (precession angle 0.6°), respectively.
